# Optimal Design of Validation Experiment for Material Deterioration

**DOI:** 10.3390/ma16175854

**Published:** 2023-08-26

**Authors:** Xiangrong Song, Dongyang Sun, Xuefeng Liang

**Affiliations:** 1 School of Naval Architecture and Ocean Engineering, Jiangsu University of Science and Technology, Zhenjiang 212003, China; xingname@163.com; 2 College of Aerospace Engineering, Chongqing University, Chongqing 400044, China; liangxuefeng2017@163.com

**Keywords:** model validation, deterioration model, validation experiment, kernel density estimation

## Abstract

For the deterioration model of a material, it is crucial to design a validation experiment to determine the ability of the deterioration model to simulate the actual deterioration process. In this paper, a design method of a validation experiment for a deterioration model is proposed to obtain the experiment scheme with low cost and satisfactory credibility. First, a normalized area metric based on probability density functions for the deterioration model is developed for validation results quantification. Normalized area metrics of different state variables in an engineering system can be applied to a unified evaluation standard. In particular, kernel density estimation is used to obtain smooth probability density functions from discrete experimental data, which can reduce the systematic error of the validation metric. Furthermore, a design method for the validation experiment for the deterioration model is proposed, in which the number of experimental samples and observation moments in each experimental sample are design variables, while the credibility of the validation experiment is the constraint. For the experiment design, the problem with varying dimensions of design variables occurred in the optimal design. Thus, a collaborative optimization method using the Latin hypercube sampling was developed to solve this problem. Finally, the results of the two examples showed the characteristics of the proposed metric and also reflected the correlation between the design variables and experimental credibility.

## 1. Introduction

High reliability and long-life materials have received increasing attention in the past and are widely used in the fields of nuclear power, aerospace, and electronic communications. As a serious concern, deterioration drastically reduces the service life and reliability of the materials. Considering the uncertainty and time-variability of the deterioration process, the deterioration is a complex and highly unpredictable process.

In recent years, there have been a lot of studies on the deterioration of materials [1]. There is generally two types of deterioration: gradual (progressive) deterioration and shock (sudden) deterioration. Gradual deterioration mainly refers to the deterioration of system parameters and performance under storage and routine use [2,3,4]. Since this deterioration phenomenon often continues over time, gradual deterioration is usually represented by a random process. Shock deterioration usually occurs in extreme cases (e.g., earthquakes, blasts and other sudden hazards) [5,6], and a point process is usually used to simulate this deterioration phenomenon. In general, the two kinds of deteriorations appear in the deterioration process of most engineering systems simultaneously. Some methods are presented for the modeling of deterioration [7,8]. Since deterioration may directly affect the reliability of the assessment results and accuracy of life prediction, deterioration models play an important role in the analysis and design of systems, especially reliability analysis [9,10], residual service life prediction [11] and life-cycle analysis [12,13].

Engineers focus on the accuracy of degradation models due to the importance of deterioration. Generally speaking, the simulation model must be verified using experimental data [14,15], and a validated deterioration model can be regarded as a credible representation of the actual deterioration phenomenon. Moreover, information gained from validation also aids in simulating the deterioration process and refining the model. Therefore, a validation approach is necessary to measure the variance between the simulation deterioration model and the experimental observations which is caused by uncertainty and model simplification. Four validation methods are prevalent in the existing literature [16,17,18,19]: classical hypothesis testing, Bayes factor, frequentist’s metric and area metric. Among them, the classical hypothesis testing judges the correctness of the model by identifying statements in which there is compelling evidence of truth. The Bayes factor verification method also pays attention to the problem of the correctness of the model [18]. Classical hypothesis testing and Bayes factor cannot obtain the variance between the simulation model and the experimental observations [17]. The frequentist’s metric used the mean of the simulation results and the experimental observations to quantitatively evaluate the difference between the simulation model and the actual model. The area metric [19] can be obtained by calculating the area between the cumulative distribution function (CDF) based on the simulation model and the CDF based on experimental data, and the size of the area can represent the variance between the simulation model and the actual model. There is a considerable amount of literature available on the validation of time-dependent models. Yang et al. [20] have proposed a validation metric of the degradation model with dynamic performance, which can not evaluate the accuracy of the degradation model quantitatively. To quantitatively evaluate the dynamic model, Zhan et al. [21] developed a Bayesian dynamic model validation method using probabilistic principal component analysis. Xi et al. [22] proposed a validation metric using U-polling techniques for general dynamic system responses. Wang et al. [23] presented an area metric based on Karhunen–Loève expansion for validating dynamic models. Atkinson et al. [24] proposed a dynamic model verification metric based on wavelet threshold signals, which can solve the problem of experimental system data being often polluted by noise. Lee et al. [25] have a detailed introduction to the above methods. In these methods, data dimensionality reduction is mainly obtained by constructing different decomposition formulas (e.g., Principal Component Analysis) to represent the entire random process. Then, the validation metric is obtained by comparing the data of the simulation model and that of the experimental observations.

For the above studies on model validation, the credibility of validation metrics is significantly reduced when the number of experimental samples is too few. However, in engineering, many experimental samples are expensive and time-consuming. This means that the credibility and cost of validation experiments are often conflicting goals. Hence, designing a validation experiment would be the key step for the validation of deterioration models. Design of experiment (DoE) [26,27] has been used to improve experimental performance in engineering and has been studied extensively. Design of experiments are classified into two categories: classical DoE (such as central composite design, full and fractional-factorial design, Box–Behnken design, orthogonal arrays experiments and optimal design) and modern DoE (such as quasi-random design, random design, projections-based design, miscellaneous design, uniform design and hybrid design). Different from traditional experiments, the validation experiment is a new type of experimental method and aims to determine the prediction accuracy and reliability of the simulation model used to describe the actual model. An excellent validation experiment can maximize the information from experiments, increase the credibility of experiments, and reduce their cost. There have been some studies on experimental design in the past decades. For example, Huan and Marzouk [28] developed an experimental design method for model calibration by using information theory metrics and gradient-based stochastic optimization techniques. Jiang and Mahadevan [29] proposed a computer simulation model verification experiment design method based on Bayesian cross-entropy. The above experimental design methods are aimed at static models and are difficult to apply to the verification of time-dependent prediction models. Ao et al. [30] proposed a validation experiment design optimization method of a life prediction model to obtain the optimal stress level and the number of experiments under each stress level. However, for the validation experiment of deterioration models, the number of experimental samples and observation moments of the samples are crucial factors in experimental design. Moreover, as an important evaluation index for validation experiments, the credibility of experiments should not be ignored. Hence, how to design the validation experiment of deterioration models under certain credibility conditions is an open problem.

This paper mainly included two main contributions: (i) A normalized area metric for deterioration models is proposed. Different from the traditional area metric, the metric is based on the probability density functions (PDFs), which is dimensionless and intuitive. In particular, kernel density estimation (KDE) is used to obtain a smooth PDF from discrete experimental data. (ii) An optimization method of the validation experiment for the deterioration model is proposed. The experimental design fully considers design variables, including the number of experimental samples, the number of observation and observation moments. The credibility of the validation experiment is constraint, and the total cost is optimization objective. This paper is structured as follows: In Section 2, the validation metric of the deterioration model is described. In Section 3, the validation experiment design of the deterioration model is developed. Finally, in Section 4, two examples of deterioration models are used to prove the correctness and validity of the experiment design method.

## 2. Validation Metric of the Degradation Model

### 2.1. Deterioration Modeling for Materials

In this work, gradual deterioration and shock deterioration are considered in the deterioration of materials. The gradual deterioration G(t) is represented using a random process, and the shock deterioration S(t) is represented using a point process. The deterioration E(t) caused by the gradual deterioration and the shock deterioration can be expressed as

(1) E(t)=[G(t),S(t)]

**Figure 1:**
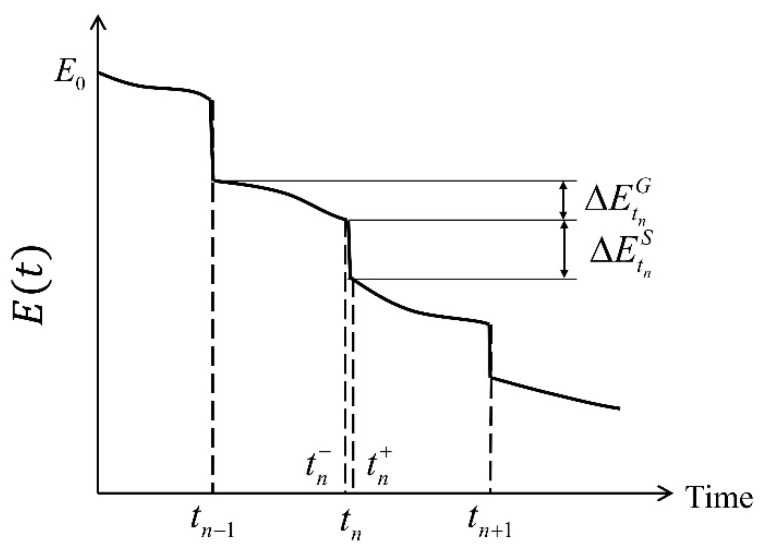
Two deterioration types: gradual deterioration and shock deterioration.

Figure 1 illustrates the deterioration process. Note that significant load events occur randomly with random intensity over time. It is shown that the shock deterioration value is $\Delta E_{t_{n}}^{S}$ at $\tau =t_{n}$, and the gradual deterioration value is $\Delta E_{t_{n}}^{G}$ in the interval $\left[t_{n-1},t_{n}\right]$. S(t) changes instantaneously from $t_{n}^{-}$ to $t_{n}^{+}$, and G(t) changes gradually from $t_{n-1}^{+}$ to $t_{n}^{-}$.

For materials, a series of state variables related to the deterioration process are available. The vector $X(t)=[X_{1}(t),X_{2}(t)\cdots X_{n}(t)]$ denotes the state variables of materials (e.g., elastic modulus of the material). For the *i*th state variable $X_{i}(t)$, its variation due to deterioration can be written as

(2) $$X_{i}(t)=X_{i,0}+\int_{0}^{t}{\dot{X}}_{i}[E(\tau ),\Theta_{i}]d\tau$$

where $X_{i,0}=X_{i}(t=0)$ is the initial state variable, and $\Theta_{i}$ is the vector of model parameters. Jia et al. [7] developed a state-dependent deterioration model, which can be defined as

(3) $$X_{i}(t)=X_{i,0}+\int_{0}^{t}{\dot{X}}_{i}[X(\tau ),E(\tau ),\Theta_{i}]d\tau$$

Compared with Equation (2), Equation (3) introduced the state dependence in deterioration, which is a versatile deterioration framework that facilitates the modeling of multiple interaction deterioration processes. For experimental data, there are measurement errors which can be expressed as $X_{i}^{e}(t)=X_{i}(t)+\varepsilon^{e}\left(t\right)$. $\varepsilon^{e}\left(t\right)$ is measurement error, which can be distinguished via multiple replications and statistical analysis.

### 2.2. Dimensionality Reduction in Deterioration Models

Since the state variables in X(t) are random processes, the degradation model obeys different distributions at different times. Thus, it is difficult to obtain a comprehensive validation metric for the degradation model using the general validation method. For the multi-response model validation, in this work, a distance formula is used for dimensionality reduction in degradation date. Let $\left\{X_{i}(t),t\geq 0\right\}$ denote the deterioration of the *i*th state variable; the distribution function of $D_{i}^{x}$ is defined as

(4) $$D_{i}^{x}=\frac{1}{\sqrt{T_{\max}}}\sqrt{\int_{0}^{T_{\max}}{\left[X_{i}^{x}(t)-\mu_{i}^{x}(t)\right]}^{T}s_{i}^{x}{\left(t\right)}^{-1}\left[X_{i}^{x}(t)-\mu_{i}^{x}(t)\right]dt}$$

where $\mu_{i}^{x}(t)$ and $s_{i}^{x}(t)$ represent the mean function and standard deviation function of the *i*th state variable, respectively. $T_{\max}$ is the maximum time of the deterioration process. Moreover, for two random processes $\left\{X_{i}(t),t\geq 0\right\}$ and $\left\{Y_{i}(t),t\geq 0\right\}$, $D_{i}^{x,y}$ can be defined as

(5) $$D_{i}^{x,y}=\frac{1}{\sqrt{T_{\max}}}\sqrt{\int_{0}^{T_{\max}}{\left[X_{i}^{x}(t)-\mu_{i}^{y}(t)\right]}^{T}s_{i}^{y}{\left(t\right)}^{-1}\left[X_{i}^{x}(t)-\mu_{i}^{y}(t)\right]dt}$$

where $\mu_{i}^{y}(t)$ and $s_{i}^{y}(t)$ represent the mean function and standard deviation function of $Y_{i}(t)$, respectively.

$D_{i}^{e,s}$ is used to capture the characteristics of the experimental data and simulation model. For the *j*th experimental data, let $\left\{D_{i,j}^{e,s},j=1,\cdots m\right\}$ follow the distribution $D_{i}^{e,s}$; it can be expressed as

(6) $$D_{i,j}^{e,s}=\frac{1}{\sqrt{o}}\sqrt{{\left[x_{i,j}^{e}-\mu_{i}^{s}\right]}^{T}{\left(s_{i}^{s}\right)}^{-1}\left[x_{i,j}^{e}-\mu_{i}^{s}\right]}$$

where *o* is the number of time points. $x_{i,j}^{e}=\left[x_{i,j}^{e}(t_{1}),x_{i,j}^{e}(t_{2})\cdots x_{i,j}^{e}(t_{o})\right]$ is the vector of the *j*th experimental data including measurement error $\mu_{i}^{s}=\left[\mu_{i}^{s}(t_{1}),\mu_{i}^{s}(t_{2})\cdots \mu_{i}^{s}(t_{o})\right]$ and $s_{i}^{s}=\left[s_{i}^{s}(t_{1}),s_{i}^{s}(t_{2})\cdots s_{i}^{s}(t_{o})\right]$ represent the mean vector and standard deviation vector of the simulation model, respectively.

The multi-dimensional distribution function is converted to the one-dimensional distance distribution. This distance distribution preserves the correlation information of each response quantity in the multi-dimensional response quantity.

### 2.3. Normalized Area Metric

**Figure 2:**
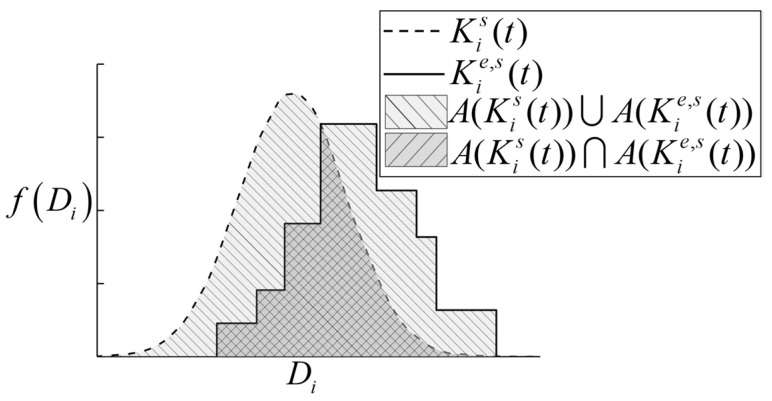
Schematic diagram of normalized area metric based on PDFs.

In this section, a normalized area metric for the deterioration model based on the PDFs is proposed to measure the variance between the deterioration model and experimental observations. Mathematically, for the *i*th state variable, the validation metric for deterioration model, as shown in Figure 2, is defined as

(7) $$\rho_{i}\left(K_{i}^{s},\;K_{i}^{e,s}\right)=\{\begin{array}{cc} 0 & I_{i}^{s}\cap I_{i}^{e,s}=\emptyset \\ \frac{A(K_{i}^{s})\cap A(K_{i}^{e,s})}{A(K_{i}^{s})\cup A(K_{i}^{e,s})} & I_{i}^{s}\cap I_{i}^{e,s}\neq \emptyset \end{array}$$

where $K_{i}^{s}$ and $K_{i}^{e,s}$ are the PDFs of $D_{i}^{s}$ and $D_{i}^{e,s}$, respectively. The superscripts *s* and *e* represent simulation model and experimental data, respectively. A(⋅) is the area between the PDF and *x*-axis. $I_{i}^{s}$ is the confidence interval for simulation result, and its range is defined as

(8) $$I_{i}^{s}=\left[\begin{array}{cc} \mu \left(D_{i}^{s}\right)-3\sigma \left(D_{i}^{s}\right) & \mu \left(D_{i}^{s}\right)+3\sigma \left(D_{i}^{s}\right) \end{array}\right]$$

$I_{i}^{e,s}$ is also an interval, and its range is defined as

(9) $$I_{i}^{e,s}=\left[\begin{array}{cc} {\underline{D}}_{i}^{e,s} & {\bar{D}}_{i}^{e,s} \end{array}\right]$$

where ${\underline{D}}_{i}^{e,s}$ and ${\bar{D}}_{i}^{e,s}$ are the lower and upper bounds of $D_{i}^{e,s}$, respectively.

There are two judgment cases: (i) If the confidence interval of simulation result and the interval of experimental data do not intersect, the simulation model is completely unreliable and $\rho_{i}\left(K_{i}^{s},\;K_{i}^{e,s}\right)=0$. (ii) If the confidence interval of simulation result intersects the interval of experimental data, a larger $\rho_{i}\left(K_{i}^{s},\;K_{i}^{e,s}\right)$ means that the simulation model is closer to the actual physical model. Furthermore, if PDFs of the simulation result and experimental data completely coincide, $\rho_{i}\left(K_{i}^{s},\;K_{i}^{e,s}\right)=1$ and the simulation model is consistent with the actual physical model. Hence, the value range of the validation metric defined using Equation (7) is [0, 1], which is an intuitive validation result that conforms to human habits of thought.

### 2.4. Kernel Density Estimation

In Equation (7), the PDF of experimental data is estimated by KDE. Let $Y_{1},\ldots ,Y_{n}\in {\mathbb{R}}^{d}$ be an independent, identically distributed random sample from an unknown distribution F with a probability density function $\hat{f}$. Mathematically, KDE can be expressed as

(10) $${\hat{f}}_{n}(y)=\frac{1}{nh^{d}}\sum_{i=1}^{n}K\left(\frac{y-Y_{i}}{h}\right)$$

where K(⋅) is a smooth function called the kernel function, h>0 is the smooth bandwidth that controls the amount of smoothing. There are many kernel functions, including uniform, triangular, biweight, triweight, Gaussian and Epanechnikov. The Gaussian kernel function is employed in this paper, which can be expressed as

(11) $$K\left(y\right)=\begin{array}{cc} \frac{\exp\left(-{\left\|y\right\|}^{2}/2\right)}{v_{d}}, & v_{d}=\int \exp \end{array}\left(-{\left\|y\right\|}^{2}/2\right)dy$$

Intuitively, the effect of KDE is to smooth each data point into a smooth bump, and the shapes of bumps are determined by the kernel function K(y). With KDE, the range of $I_{i}^{e,s}$ in Equation (7) can be redefined as

(12) $$I_{i}^{e,s}=\left[\begin{array}{cc} \mu \left(D_{i}^{e,s}\right)-3\sigma \left(D_{i}^{e,s}\right) & \mu \left(D_{i}^{e,s}\right)+3\sigma \left(D_{i}^{e,s}\right) \end{array}\right]$$

## 3. Validation Experiment Design for Deterioration Models

In the engineering applications of deterioration models, the validation metric is directly related to the number of validation experiments, and the credibility of the validation metric is significantly reduced if the number of experimental samples is too few. However, since deterioration models are often used for products with high reliability and long life, the experimental samples of deterioration models are generally time-consuming and expensive. Obtaining validation experiments with high credibility and reducing experimental costs are conflicting goals. To obtain the validation experiment scheme for deterioration models with low cost and satisfactory credibility, in this section, an optimal design method of validation experiment is developed.

### 3.1. Optimization Model of Validation Experiment for Deterioration Models

In engineering, researchers usually focus on obtaining a qualitative validation result for deterioration models. Similarly, for the validation experiment of deterioration models, evaluating the validation results of the deterioration models is an important step. Considering the value range of normalized area metric for deterioration models, an evaluation standard is established to qualitatively evaluate validation results, as shown in Table 1. For the *i*th state variable, the value of $\phi_{i}$ in Table 1 can be identified by the specific validation experiments and expert experience.

For the validation experimental design of deterioration models, three variables that affect the experimental results should be considered in this work: the number of experimental samples *m*, the number of experimental observations *o*, and the vector of observation moments $T_{o}=\left[T_{1},\;T_{2},\;\cdots T_{o}\right]$. Ideally, the actual deterioration model can be reflected by enough experimental samples, but it leads to expensive experimental costs. For the second variable *o*, although enough observational data can increase the accuracy of validation experiments, expensive observation cost is also unacceptable for engineers. In the third variable $T_{o}$, the improvement of experimental quality is effective through a reasonable allocation of observation moments. Therefore, in this work, the above three variables are used as the design variables of the validation experiments. Considering that the credibility of validation experiments and the experimental cost are conflicting goals, the optimization model of validation experiments can be written as

(13) $$\begin{array}{ll} \min & T_{C}\left(m,o,T_{o}\right)\\ s.t. & P\left(\rho_{i}\left(K_{i}^{s},\;K_{i}^{e,s}\left(m,o,T_{o}\right)\right)\geq \phi_{i}^{III}\right)\geq P_{r}\\ & m_{\min}\leq m\leq m_{\max}\\ & o_{\min}\leq o\leq o_{\max}\\ & 0\leq T_{i}\leq T_{\max} \end{array}$$

where $P_{r}$ is the pre-set threshold that is used to evaluate the credibility of validation experiments, and $m_{\min}$ and $m_{\max}$ are the minimum and maximum design number of experimental samples, respectively. $o_{\min}$ and $o_{\max}$ denote the minimum and maximum number of observation instants, respectively. $T_{\max}$ is the maximum experimental observation instant. P(⋅) is a probability function used to count the proportion of validation experiments of “excellent”. $T_{C}$ is the total cost, which is obtained via

(14) $$T_{C}\left(m,o,T_{o}\right)=C_{u}\cdot m+C_{p}\cdot m\cdot \max\left(T_{i}\right)+C_{m}\cdot m\cdot o$$

where $C_{u}$ is the cost of an experimental sample, $C_{p}$ is the labor cost and public resource cost to maintain the experimental environment in a unit time and $C_{m}$ is the experimental cost of one validation observations.

### 3.2. Design Process for Validation Experiments

**Figure 3:**
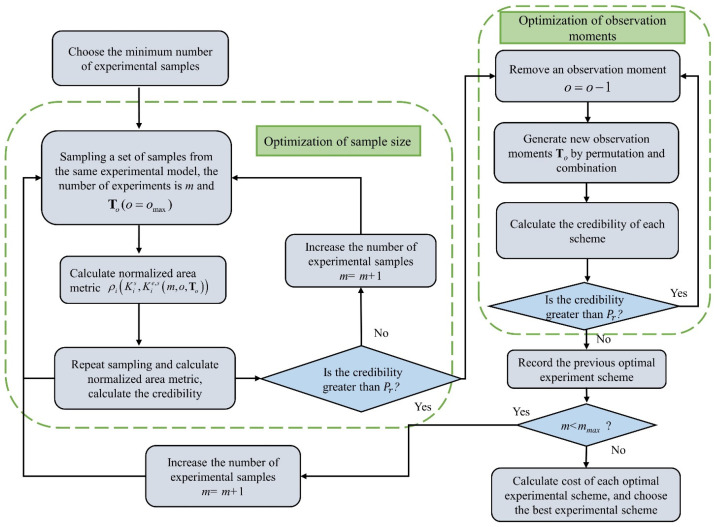
Flowchart of validation experiment design.

For the above optimization problem, $T_{o}$ and o are related to each other, and the dimension of the vector $T_{o}$ is determined by o. Therefore, two problems need to be solved in the optimization process: the varying dimensions of design variables and the non-independent relationship of design variables in the optimization process. General optimization methods have difficulties solving Equation (13). Considering that the number of experimental samples has the most significant impact on the total cost and credibility, a collaborative optimization method was used in this study. In which, the number of experimental samples is the first value to be individually optimized, then o and $T_{o}$ are optimized after the number of samples is determined. The main procedures of the method are shown in Figure 3 with the following steps.

**Optimization of the number of experimental samples:**

The optimization model can be written as

(15) $$\begin{array}{ll} \min & T_{C}\left(m,o_{\max},T_{o_{\max}}\right)\\ s.t. & P\left(\rho_{i}\left(K_{i}^{s},\;K_{i}^{e,s}\left(m,o_{\max},T_{o_{\max}}\right)\right)\geq \phi_{i}^{III}\right)\geq P_{r}\\ & m_{\min}\leq m\leq m_{\max} \end{array}$$

The procedures of the optimization are:

Step 1: Choose the minimum number of experimental samples $m=m_{\min}$, the number of observation instants $o=o_{\max}$ and the observation moments $T_{o}=T_{o_{\max}}$.
Step 2: Sampling a set of samples from the same experimental model, the number of experiments is *m* and $T_{o}(o=o_{\max})$.
Step 3: Calculate the normalized area metric $\rho_{i}\left(K_{i}^{s},\;K_{i}^{e,s}\left(m,o_{\max},T_{o_{\max}}\right)\right)$.
Step 4: Repeat Steps 2–3 and calculate the credibility $P\left(\rho_{i}\left(K_{i}^{s},\;K_{i}^{e,s}\left(m,o_{\max},T_{o_{\max}}\right)\right)\geq \phi_{i}^{III}\right)$ of the current scheme.
Step 5: If $P\left(\rho_{i}\left(K_{i}^{s},\;K_{i}^{e,s}\left(m,o_{\max},T_{o_{\max}}\right)\right)\geq \phi_{i}^{III}\right)\geq P_{r}$, go optimize observation instants. If not, increase the number of experimental samples *m = m +* 1 and repeat Steps 2–5.

**Optimization of the observation instants:**

The optimization model can be written as

(16) $$\begin{array}{ll} \min & T_{C}\left(m,o,T_{o}\right)\\ s.t. & P\left(\rho_{i}\left(K_{i}^{s},\;K_{i}^{e,s}\left(m,o,T_{o}\right)\right)\geq \phi_{i}^{III}\right)\geq P_{r}\\ & o_{\min}\leq o\leq o_{\max}\\ & 0\leq T_{i}\leq T_{\max} \end{array}$$

The procedures of the optimization are:

Step 6: The number of experimental samples is obtained via Equation (15). Remove an observation moment under the current experimental sample size: o=o−1.
Step 7: Generate new observation moments $T_{o}$ by permutation and combination and calculate the credibility.
Step 8: Calculate the credibility of each scheme.
Step 9: If $P\left(\rho_{i}\left(K_{i}^{s},\;K_{i}^{e,s}\left(m,o,T_{o}\right)\right)\geq \phi_{i}^{III}\right)\geq P_{r}$, continue to remove an observation moment and repeat Steps 7–9. If not, record the previous optimal scheme and go to Step 10.
Step 10: Determine whether the current sample size is less than the maximum number of experimental samples $m_{\max}$. If Yes, increase the number of experimental samples *m = m +* 1 and go to Step 2. If No, go to Step 11.
Step 11: Calculate the cost of each optimal experimental scheme and choose the best experimental scheme.

## 4. Results and Discussion

This section presents two examples to illustrate the proposed method. The first one is a mathematical model that focuses on the validation metric for deterioration models. The second one is an engineering example of a B-pillar that focuses on the validation experimental design for deterioration models.

### 4.1. Mathematical Model

Considering the time-related state variables caused by deterioration, a random process model that can be used for deterioration is proposed. For an engineering system, a simulation model of state variable can be expressed as

(17) $$y^{s}=\theta bt+S(t)$$

(18) $$\theta =\theta_{0}(1+e^{at})$$

where $\theta_{0}$ is a constant parameter of the engineering system, $\theta_{0}=1$. *a* and *b* are the uncertain parameters, and both are the normal distribution, $a\sim N(0.02,0.005^{2})$ and $b∼N(1.0.1^{2})$. S(t) is the stationary Poisson process with λ=1. For this actual deterioration model, Table 2 proposes five actual deterioration models from the experiments. It can be seen that only Model 1 is consistent with the simulation model, the constant parameter $\theta_{0}$ of Model 2 is different to the simulation model, the different parameters of Model 3 and Model 4 are a and λ, respectively, and three parameters of Model 5 are different to the simulation model.

**Figure 4:**
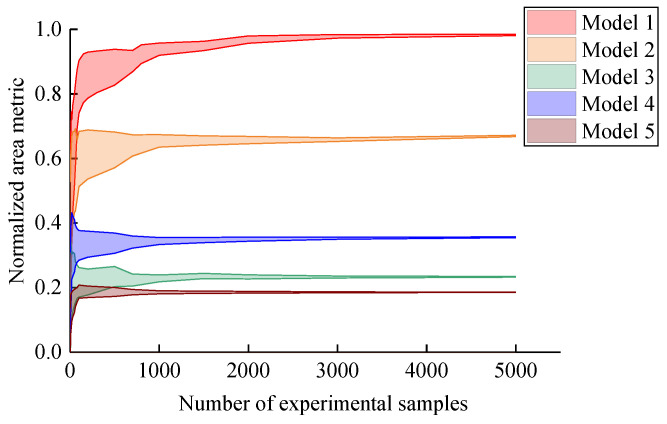
Normalized area metrics with number of experimental samples.

The normalized area metrics between five actual deterioration models and the simulation deterioration model are calculated, respectively. Figure 4 presents the changing trend of the normalized area metrics with the number of experimental samples. It can be found that five metrics of deterioration models gradually converge with the increase in sample size. The convergence value can truly reflect the validation result of each model. For metrics in the convergence results, Model 1 is better than Model 2, Model 2 is better than Model 4, Model 4 is better than Model 3 and Model 5 is the worst. The results reflect the effect of different parameters in Table 1 on the metrics of deterioration models. The metric of Model 1 tends to 1 because it is consistent with the simulation model. Since the three parameters of Model 5 are different from the simulation model, the convergence value of the metric is the smallest. Moreover, the range of five metrics also decreases with the increase in the number of experimental samples. Thus, the actual probability distribution can be reflected by enough samples.

**Figure 5:**
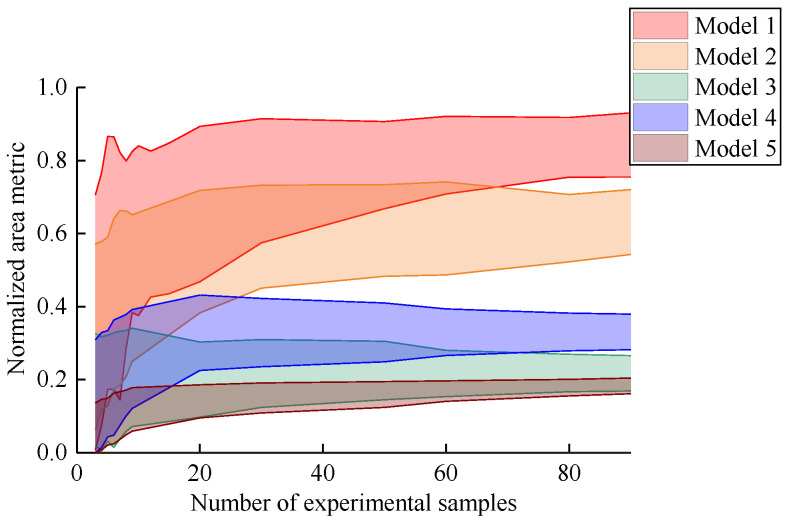
Normalized area metrics in the small sample.

In the case of small samples, as shown in Figure 5, it can be found that the five metrics have significant errors with regard to the final convergence values. Furthermore, the metrics of the five models have overlapping areas. Thus, the evaluation of the deterioration model may not be true in this case. In summary, in the case of small samples, the normalized area metric for deterioration models has a greater dispersion, which leads to the model evaluation results not being credible. On the contrary, the dispersion of the normalized area metric decreases with the increase in sample size, which leads to the improvement of the credibility of the model evaluation results.

**Figure 6:**
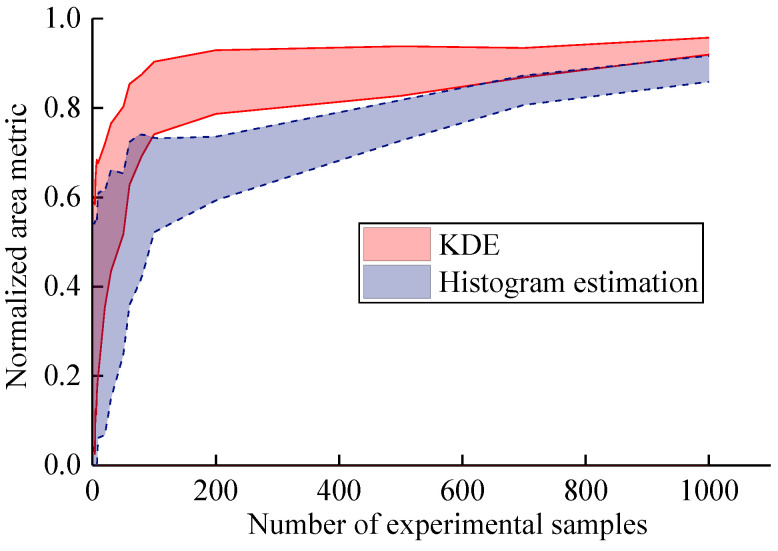
Normalized area metrics based on KDE and histogram estimation.

In particular, KDE replaces histogram estimation to estimate discrete experimental data in this work. In order to understand the effect of KDE, Figure 6 presents the normalized area metrics based on KDE and histogram estimation (the example is Model 1). It can be seen that the convergence speed of KDE method is faster than the histogram estimation method. Especially in small samples, the unsmooth histogram leads to larger validation errors. For enough experimental samples, the PDFs from the histogram estimation tend to be smooth, and the errors of the two methods are gradually smaller. For small sample cases, KDE has more advantages compared to histogram estimation, which is more suitable for the validation experimental design in this work.

### 4.2. Cantilever Beam

**Figure 7:**
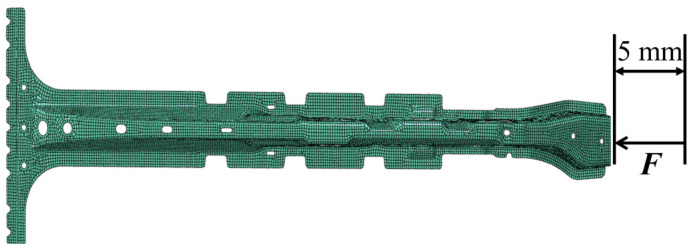
Finite element model of B-pillar.

The second example is a B-pillar in cars that focuses on validation experimental design for deterioration models. The B-pillar used shell elements for finite element modeling, and its finite element model is shown in Figure 7. There are 13,582 elements and 23,326 nodes in the entire model. The B pillar is made of hot forming steel. Hot formed steel is a homogeneous isotropic material, and the deterioration model of its elastic modulus is as follows:

(19) $$E(t)=E_{0}(1-D(t))$$

where $E_{0}=206\;\mathrm{GPa}$ is the initial modulus, D(t) represents the deterioration process, and the maximum design time $T_{\max}$ is 40 years for this experiment. For the deterioration process D(t), gradual deterioration and shock deterioration are considered. The gradual deterioration G(t) is modeled via a Gamma process; the mean value of the cumulative gradual deterioration varies linearly with time and reaches $0.2E_{0}$ over a service period of $T_{\max}$ (the average deterioration rate $\alpha_{g}=0.005/\mathrm{year}$), and the covariance (COV) of the cumulative gradual deterioration at $T_{\max}$ is 0.3. The shock occurrence S(t) is modeled by a Poisson process with a mean occurrence rate of $\lambda_{s}=0.2/\mathrm{year}$, and each shock event independently follows a lognormal distribution with a mean value of 0.02 and a COV of 0.05. In this work, the compression experiment of the B-pillar is employed for observation. In the experiment, the left end of the B-pillar is welded to the car’s floor pan; the force from the testing machine is applied at the right end, and the reaction force is recorded as a state variable when the displacement at the right end is 5 mm.

#### 4.2.1. Optimization of Validation Experiments

According to the method in Section 3.2, Table 3 presents the relevant parameters for validation experiment design of the B-pillar, where experimental data can be obtained after one year. By using the optimization method for the validation experiment, as shown in Figure 3, three optimization schemes are listed in Table 4. It is visible that in order to acquire the credibility of the validation experiment, the minimum number of experimental samples is 65, in which all experimental data are necessary. $P\left(\rho_{i}\left(K_{i}^{s},\;K_{i}^{e,s}\left(m,o,T_{o}\right)\right)\geq 0.75\right)$ of three experimental schemes are 80.4%, 80.9% and 80.1%, respectively. The comparison between Schemes 1 and 2 shows that the $P\left(\rho_{i}\left(K_{i}^{s},\;K_{i}^{e,s}\left(m,o,T_{o}\right)\right)\geq 0.75\right)$ of the validation experiment increases with the increase in experimental samples, this trend is consistent with the characteristics of normalized area metrics in the previous mathematical example. Thus, the number of experimental samples has a significant impact on the credibility of validation experiment, and a credible validation experiment requires a certain number of experimental samples.

In particular, by using the proposed optimization method, the lowest cost one is Scheme 3 in three experimental schemes. Although the sample size of Scheme 3 is larger than that of Scheme 1, the cost of Scheme 3 is lower due to the fewer observation moments. The collaborative optimization method is used to obtain the potential optimization result in this work. The experimental schemes with fewer observation moments and more experimental samples may also be the best candidates for the validation experiment, especially when the observation cost is higher than the sample cost.

#### 4.2.2. Impact of Dispersion and Observation Moments on Credibility

**Figure 8:**
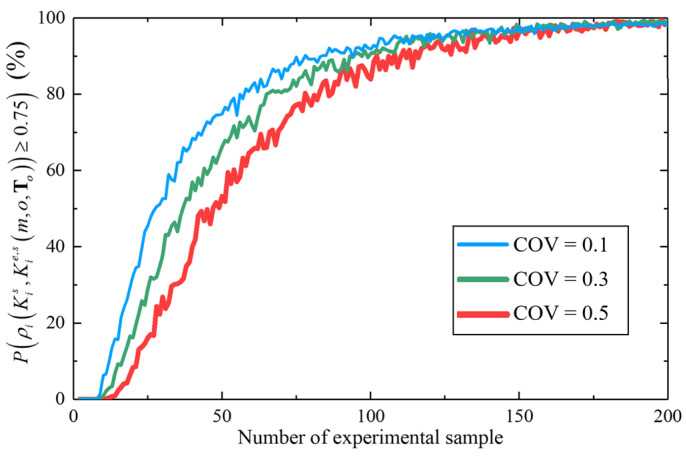
$P\left(\rho_{i}\left(K_{i}^{s},\;K_{i}^{e,s}\left(m,o,T_{o}\right)\right)\geq 0.75\right)$ with the number of experimental samples in different COVs.

To investigate the impact of dispersion of deterioration processes on the credibility of the validation experiment, Figure 8 shows the changing trend of $P\left(\rho_{i}\left(K_{i}^{s},\;K_{i}^{e,s}\left(m,o,T_{o}\right)\right)\geq 0.75\right)$ with the number of experimental samples (from 2 to 200) in different COVs associated with the cumulative gradual deterioration over 40 years. It can be found that the general change trend of $P\left(\rho_{i}\left(K_{i}^{s},\;K_{i}^{e,s}\left(m,o,T_{o}\right)\right)\geq 0.75\right)$ is increasing, converging to 100% with the number of experimental samples. In addition, the distribution function of experimental data is significantly affected by random sampling in the case of a small sample, which eventually leads to the small fluctuation of the curve. More importantly, for three different COVs, the smaller the value of COV, the faster the convergence speed of curve. The reason is that the larger COV represents the greater dispersion in the deterioration process, and only more sample data can correctly describe the probability distribution. For example, in the case of “COV = 0.1”, the experimental credibility reaches 80.7% when the sample size is 56, but in the case of “COV = 0.5”, when the sample size is 78, the experimental credibility can reach 80.2%.

To understand the influence of observation moments on the credibility of experimental schemes, we analyzed the changing trend of the credibility with the observation moments for the case of “COV = 0.3”. For the observation moments, when the number of experimental samples is 66, the changing trend of credibility with the reduced number of observation moments is listed in Table 5. It is seen that when the number of experimental samples is 66, the observation scheme that subtracts one observation moment has less impact on the maximum credibility. However, the credibility is significantly reduced when two or more observation moments are subtracted. Based on the above content, the experimental sample size and detection time of the validation experiment are both important design parameters for experimental credibility.

## 5. Conclusions

This paper proposed a design method for a validation experiment for material deterioration to obtain an experiment scheme with low cost and satisfactory credibility, in which the proposed normalized area metrics are suitable for the validation of versatile deterioration models. The number of experimental samples and the observation moments are crucial factors for experimental design, and the credibility of validation experiments is used as a constraint. Two simulation examples are adopted to validate the proposed methods in this paper, in which a simple mathematical example illustrated that the validation metric of deterioration models gradually converges with the increase in the sample size, and the validation results may be false in the case of small sample sizes. Similarly, in the case of small samples, KDE has more advantages compared to histogram estimation. Another engineering example illustrated the implementation of the proposed optimization framework. The results showed that although the number of experimental samples has a significant impact on the credibility and cost of the experiment, the experimental schemes with fewer observation moments and more experimental samples may also be the best candidates for validation experiments. Both the number of experimental samples and the detection moments are related to the credibility of the validation experiment. These two factors are essential for experiment schemes with low cost and satisfactory credibility.

## Figures and Tables

**Table 1:** Validation result based on the validation metric for deterioration models.

Validation Metric	$0\leq \rho_{i}<\phi_{i}^{I}$	$\phi_{i}^{I}\leq \rho_{i}<\phi_{i}^{\mathrm{II}}$	$\phi_{i}^{\mathrm{II}}\leq \rho_{i}<\phi_{i}^{\mathrm{III}}$	$\phi_{i}^{\mathrm{III}}\leq \rho_{i}<1$
validation result	worst	moderate	good	excellent

**Table 2:** Deterioration parameters of five models.

	$\theta_{0}$	a	λ
Model 1	1	$N(0.02,0.005^{2})$	1
Model 2	1.2	$N(0.02,0.005^{2})$	1
Model 3	1	$N(0.0015,0.01^{2})$	1
Model 4	1	$N(0.02,0.005^{2})$	2
Model 5	1.2	$N(0.0015,0.01^{2})$	2

**Table 3:** Parameters in the validation experiment design of B-pillar.

Parameter	Value	Parameter	Value
$\phi^{III}$	0.75	$P_{r}$	80%
$C_{u}$	$16	$C_{p}$	$2/year
$C_{m}$	$20.5		

**Table 4:** Optional optimization schemes of validation experiments.

Scheme	Number of Experimental Samples	Reduced Number of Observations	Total Cost	*P*
1	65	0	$59,540	80.4%
2	66	0	$60,456	80.9%
3	66	1 (32nd year)	$59,103	80.1%

**Table 5:** Changing trend of credibility with the reduced number of observation moments.

Reduced Number of Observation Moments	0	1	2	3	4
P	80.9%	80.1%	66.8%	53.4%	35.8%

## Data Availability

Not applicable.

## Abbreviations

**Nomenclature**			
X	degradation variable	t	degradation time
D	distance	μ	mean value
s	standard deviation	ρ	normalized area metric
A	the area between function and *x*-axis	K	probability density function
I	interval number	$T_{o}$	vector of observation instants
*o*	number of observation instants	*m*	number of experimental samples
$P_{r}$	pre-set threshold	P	probability function
$T_{C}$	total cost of validation experiment	$C_{u}$	cost of an experimental sample
$C_{m}$	cost of an experimental observation	$C_{p}$	labor cost and public resource cost of a sample in a unit time
**Subscript**			
*e*	experimental data	*s*	simulation model
$\underline{}$	lower bound of interval number	$\bar{}$	upper bound of interval number
